# Supplementary material for: Inclusion of variants discovered from diverse populations improves polygenic risk score transferability
Source: HGG Adv. 2020 Dec 2;2(1):100017. doi: 10.1016/j.xhgg.2020.100017 (PMC7869832; doi:10.1016/j.xhgg.2020.100017)
Supplement: Document S1. Figures S1–S6 and Tables S1 and S2 [file mmc1.pdf]

XHGG, Volume *2*

Supplemental Information

Inclusion of variants discovered from diverse populations improves polygenic risk score transferability

Taylor B. Cavazos and John S. Witte

SUPPLEMENTAL MATERIALS

Figure S1. European Derived Risk Score Accuracy with Varying Simulation Causal Variants and Assumed Heritability

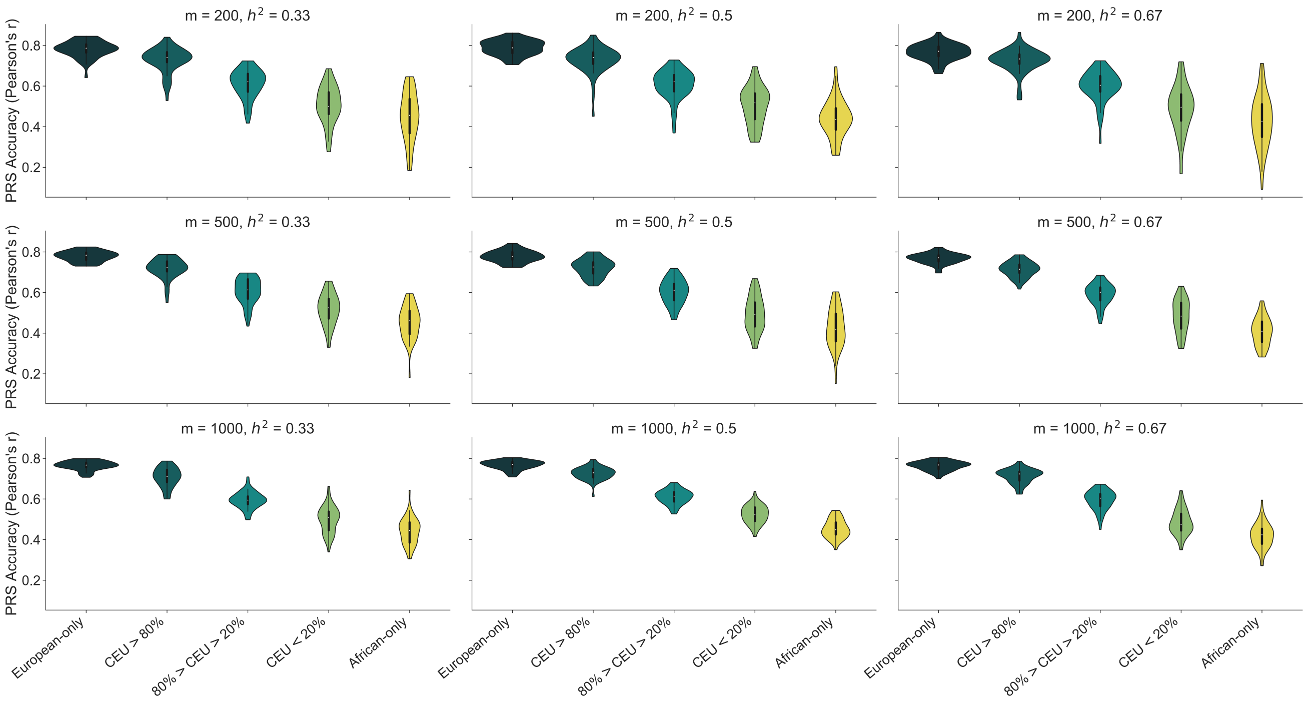

Figure S1 Legend: Our simulation assumes a set number of causal variants (m) and trait heritability (h^2) when generating the true genetic risk score. We varied these parameters and tested all combinations assuming m = {200, 500, 1000} and h^2 = {0.33, 0.5, 0.67}. The accuracy was measured by Pearson's correlation between the true and GWAS estimated risk score for each of the 50 simulations. We used a European GWAS to select independent variants and effect sizes for the PRS ($p < 0.01$ and LD $r^2 = 0.2$). This risk score was applied to Europeans, Africans, and admixed populations.

Figure S2. PRS Accuracy with Varying P-value Thresholds

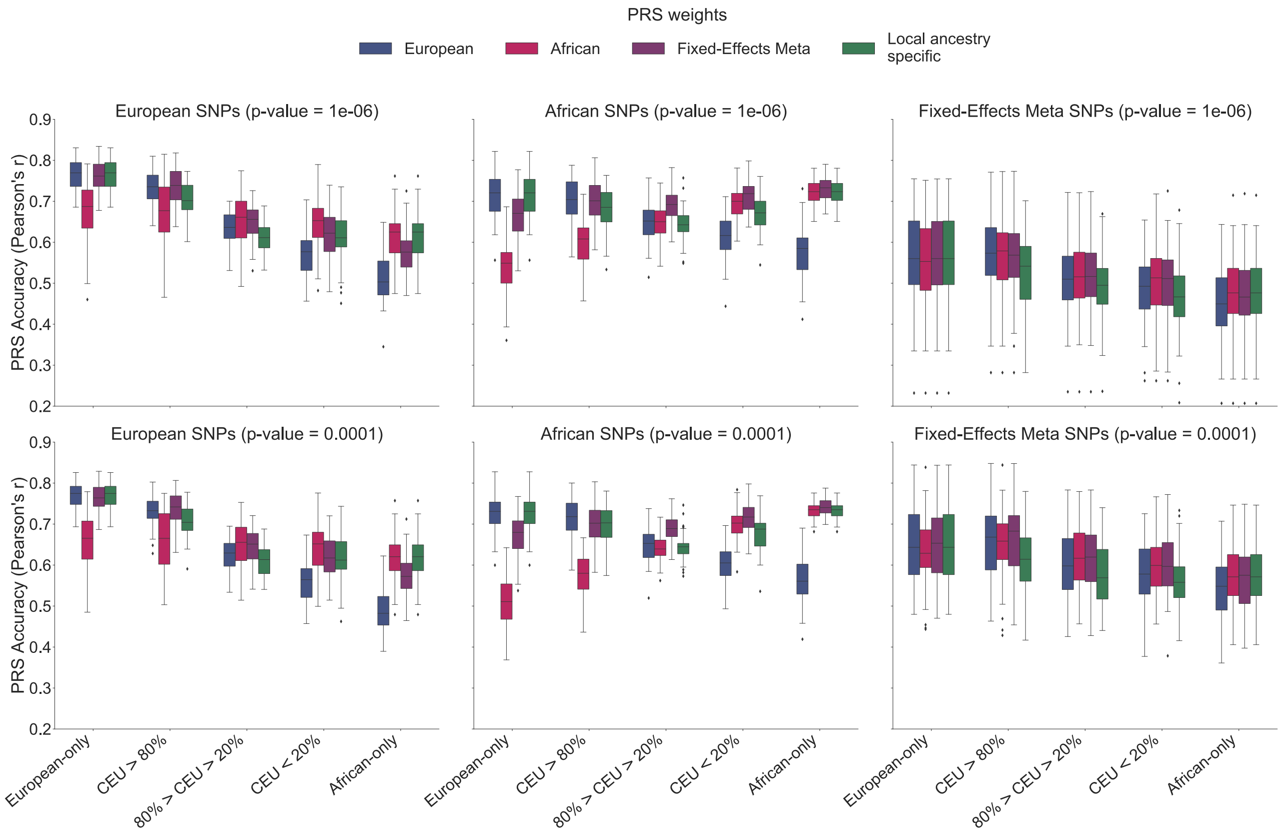

Figure S2 Legend: The p-value used for variant selection approach was decreased to allow fewer variants into the PRS. In addition to p < 0.01, shown in the main text results, we also tested p < $1x10^{-4}$ and $1x10^{-6}$. Simulations assume 1000 causal variants and a heritability of 0.5 to compute the true genetic risk. A p-value of 0.01 and LD r^2 cutoff of 0.2 was used to select variants for the estimated risk scores.

Figure S3. Total Liability Variance Explained by a European Derived Risk Score as a Function of Genetic Admixture with Varying Simulation Parameters

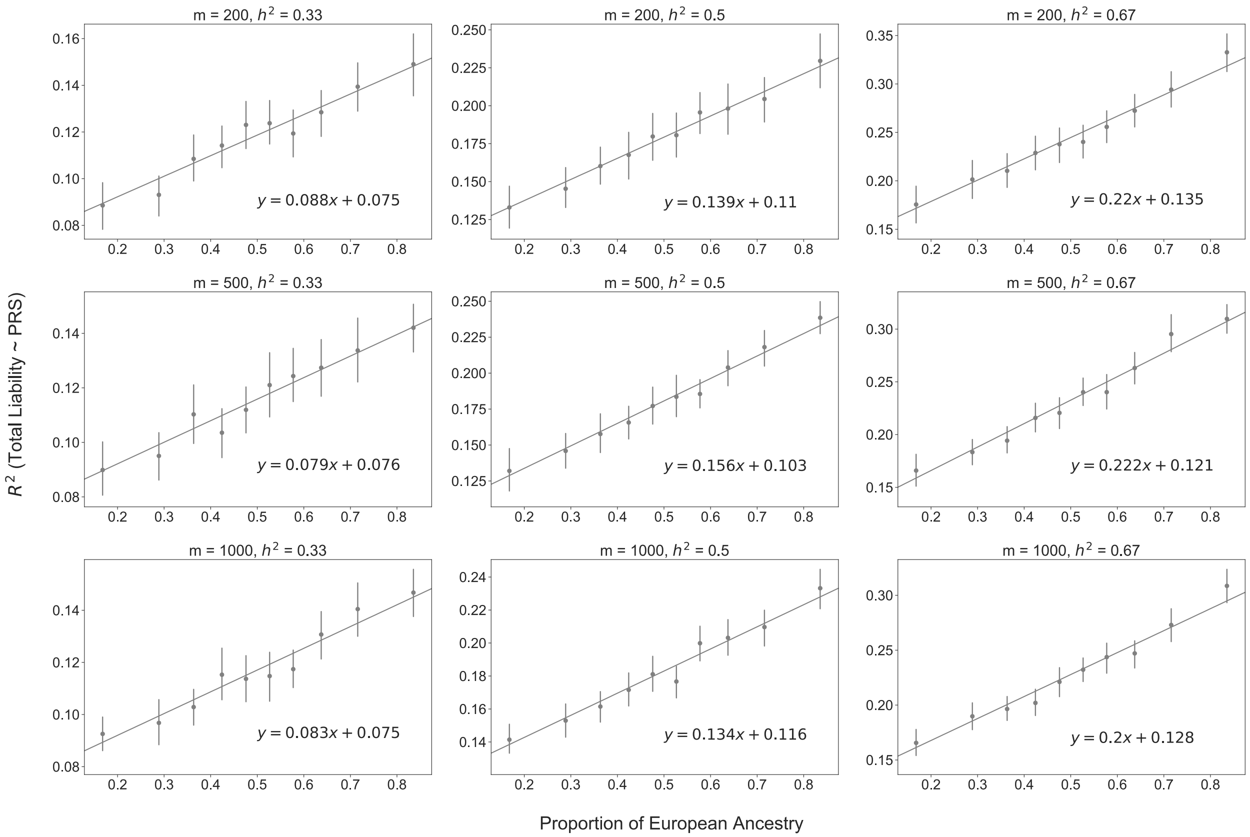

Figure S3 Legend: Admixed individuals were split into deciles of global genome-wide European ancestry. For each simulation trial the variance explained (R^2) of the total trait liability (genetics + environment) by the estimated PRS (European variants and weights) was computed within each ancestry decile. Across simulations the average ancestry for each bin is reflected as the point and the vertical bar represents the 95% confidence intervals. The equation shows the slope of each line.

Figure S4: Linear Mixture of Multiple Population Derived PRS Removes Bias Between Accuracy and Ancestry

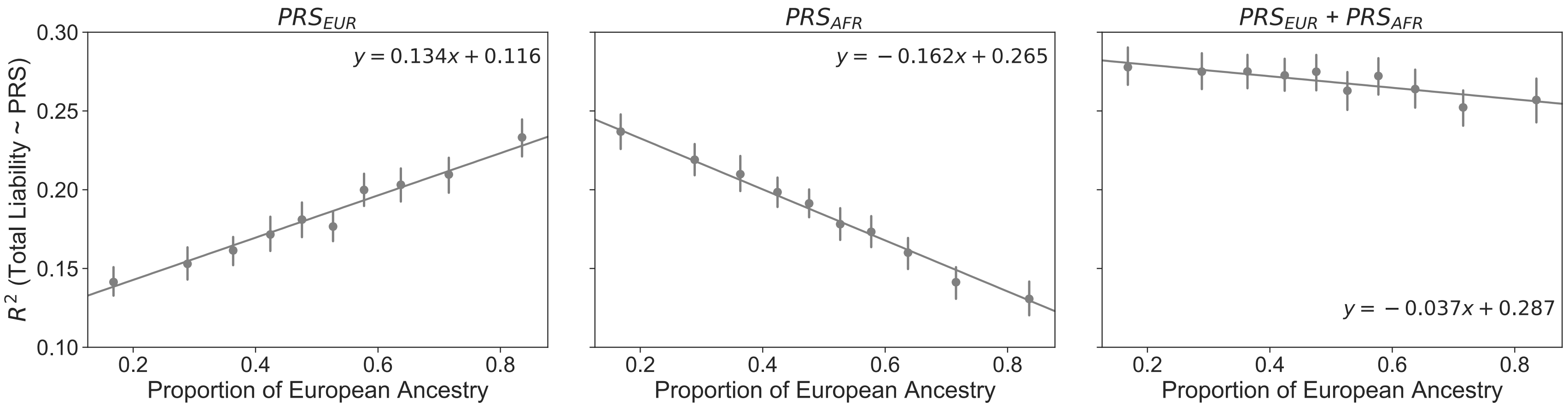

Figure S4 Legend: Two population derived risk scores (PRS_{EUR} and PRS_{AFR}) were constructed from variants and weights from GWAS in the designated simulated population and applied to an independent simulated admixed population. The PRS were combined ($\alpha_1 PRS_{EUR} + \alpha_2 PRS_{AFR}$) through a linear mixture approach described by Márquez-Luna et. al.[1] where the mixing coefficients (α_1 and α_2) were estimated in an independent African ancestry testing population and validated in the admixed population. Admixed individuals were split into deciles of global genome-wide European ancestry and for each simulation trial variance explained (R^2) of the total trait liability by each PRS approach was calculated within each decile. Across simulations the average ancestry for each bin is reflected as the point and the vertical bar represents the 95% confidence intervals. The equation shows the slope of each line.

Figure S5. Normalized LD Tagging of Causal Variants by GWAS Selected Variants

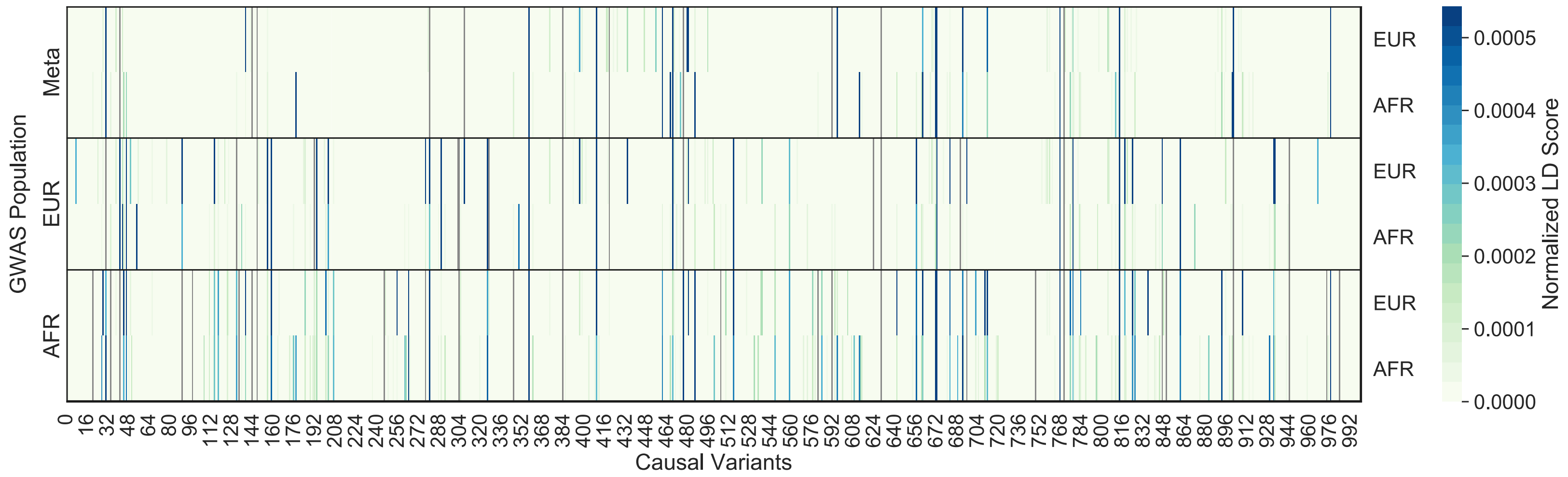

Figure S5 Legend: LD scores were calculated for every causal variant by adding up the LD r^2 for each GWAS tag variant within ±1000 kb of the causal variant. LD scores were then normalized by the total number of GWAS selected variants from each population reflected by the 3 panels. The normalized LD scores for each causal variant (m = 1000) calculated in a European or African population is shown for one simulation. Causal variants directly discovered through the GWAS are colored in gr

Figure S6: PRS Performance Across SNP Selection and Weighting Approaches for Multiple Complex Traits in the UK Biobank

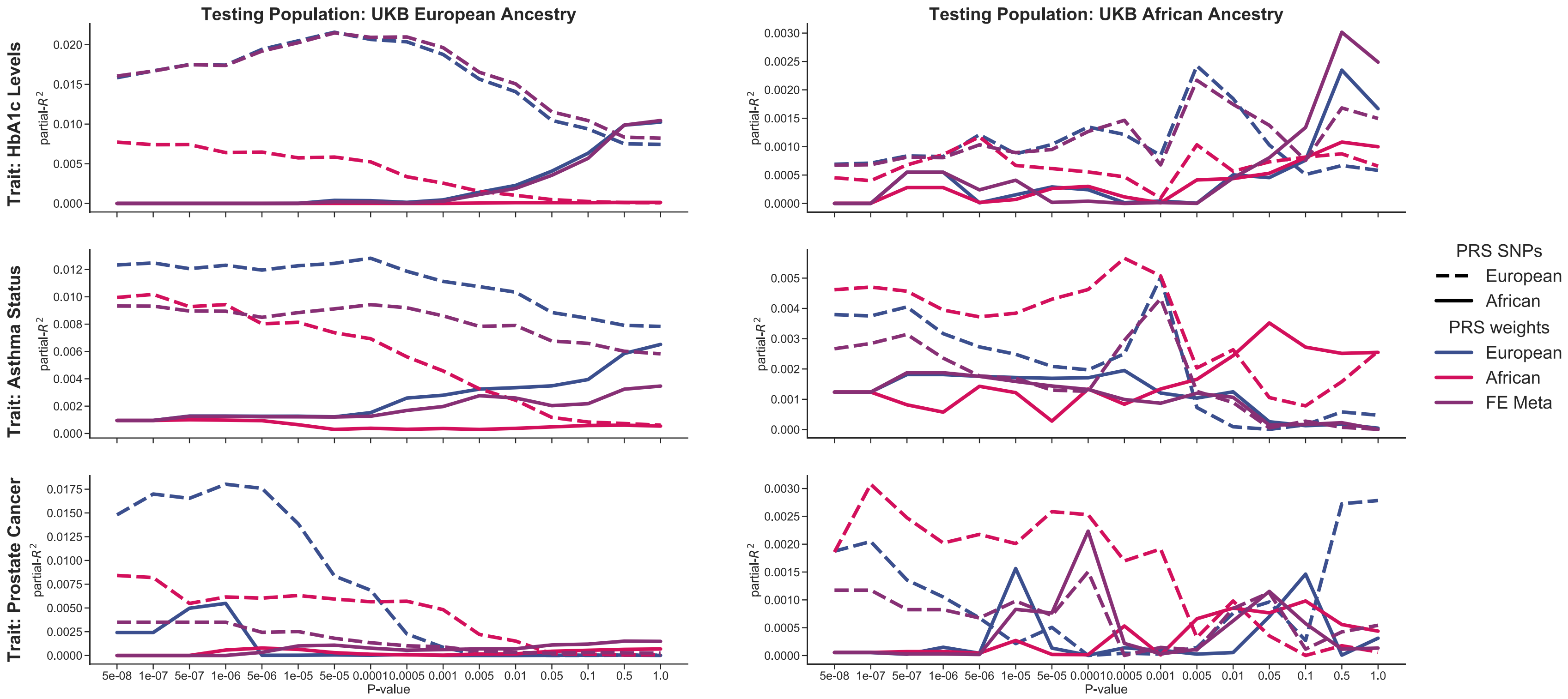

Figure S6 Legend: Partial-R^2 was calculated for each PRS, within Europeans and Africans from the UK Biobank, by subtracting the variance explained by the null model. For HbA1c we fit a linear model with age, sex, BMI, and PCs1-10 with and without the PRS. Similarly for asthma status and prostate cancer, we determined the Nagelkerke's pseudo partial-R2 following logistic regression of case status on age, sex (asthma only), BMI (prostate cancer only), and PCs1-10 with and without the PRS.

Table S1. Independent GWAS Summary Statistics and UK Biobank Testing Population used for PRS

Trait	Study	# Variants	# Samples Summary Statistics (cases)		# Samples Testing – UK Biobank (cases)	
			European Ancestry	African Ancestry	European Ancestry	African Ancestry
HbA1c	Wheeler et al. 2017[2]	1,768,940	123,665 (NA)	-	395,472 (NA)	5,886 (NA)
	Wheeler et al. 2017[2]	1,545,588	-	7,564 (NA)		
Asthma	Demenais et al. 2018[3]	1,202,829	127,669 (19,954)	–	413,870 (54,537)	7,250 (1,002)
	Daya et al. 2019[4]	12,955,021	–	14,654 (7,009)		
Prostate Cancer	Emami et al. 2020[5] (KP RPGEH)	21,230,454	11,649 (6,196)	–	413,870 (7,610)	7,250 (166)
	Conti et al. 2020[6] (AAPC)	9,910,837	–	9,531(4,853)		

Table S1 Legend: Description of cohort sizes of independent summary statistics used for PRS construction and UK Biobank population used for assessing PRS accuracy. Case counts are not applicable (NA) for non-binary traits. For prostate cancer, GWAS studies included multiple populations; however, we only used the Kaiser Permanente Research Program in Genes, Environment, and Health (KP RPGEH) summary statistics from *Emami et al. 2020* and the African American Prostate Cancer (AAPC) consortium data from *Conti et al. 2020*. Number of variants from each independent study that were available for analysis in both testing populations from the UK Biobank are reported here.

Table S2. Cross-Validated Accuracy for a Linear Combination of Multiple PRS in the UKB African Ancestry Population

Trait	Best PRS – African Selected SNPs				Best PRS – European Selected SNPs				Combined PRS
	Weights	P-value	# SNPs	Partial-R^2	Weights	P-value	# SNPs	Partial-R^2	Partial-R^2
HbA1c	FE Meta	0.5	146,670	0.0040	European	$5x10^{-3}$	2,538	0.0022	0.0052
Asthma	African	0.05	157,573	0.0036	African	$5x10^{-4}$	556	0.0060	0.0089
Prostate Cancer	FE Meta	$1x10^{-4}$	190	0.0047	African	$1x10^{-7}$	10	0.0082	0.0129

Table S2 Legend: The PRS with the highest Partial-R^2 in the full UKB African ancestry testing cohort was chosen using African ancestry or European ancestry selected SNPs from independent summary statistics for each trait. A combined PRS was constructed by using a linear combination of the two best PRS ($\alpha_1 PRS_{EUR} + \alpha_2 PRS_{AFR}$). Through 5-fold cross validation, 80% of the cohort was used to estimate the mixing coefficients (α_1 and α_2) and the accuracy of combined PRS was tested in the remaining 20% of the cohort. For disease traits, stratified 5-fold cross validation was used to ensure a consistent ratio of cases and controls across folds. Accuracy was reported as the partial-R^2 or the proportion of variation explained by the PRS. Accuracy of the single PRS were also re-assessed through 5-fold cross validation for comparison.

SUPPLEMENTAL REFERENCES

1. Márquez-Luna, C., Loh, P.-R., and Price, A.L. (2017). Multi-ethnic polygenic risk scores improve risk prediction in diverse populations. Genet Epidemiol *41*, 811–823.

2. Wheeler, E., Leong, A., Liu, C.-T., Hivert, M.-F., Strawbridge, R.J., Podmore, C., Li, M., Yao, J., Sim, X., Hong, J., et al. (2017). Impact of common genetic determinants of Hemoglobin A1c on type 2 diabetes risk and diagnosis in ancestrally diverse populations: A transethnic genome-wide meta-analysis. PLoS Med *14*, e1002383.

3. Australian Asthma Genetics Consortium (AAGC) collaborators, Demenais, F., Margaritte-Jeannin, P., Barnes, K.C., Cookson, W.O.C., Altmüller, J., Ang, W., Barr, R.G., Beaty, T.H., Becker, A.B., et al. (2018). Multiancestry association study identifies new asthma risk loci that colocalize with immune-cell enhancer marks. Nat Genet *50*, 42–53.

4. CAAPA, Daya, M., Rafaels, N., Brunetti, T.M., Chavan, S., Levin, A.M., Shetty, A., Gignoux, C.R., Boorgula, M.P., Wojcik, G., et al. (2019). Association study in African-admixed populations across the Americas recapitulates asthma risk loci in non-African populations. Nat Commun *10*, 880.

5. Emami, N.C., Cavazos, T.B., Rashkin, S.R., Cario, C.L., Graff, R.E., Tai, C.G., Mefford, J.A., Kachuri, L., Wan, E., Wong, S., et al. (2020). Association Study of Over 200,000 Subjects Detects Novel Rare Variants, Functional Elements, and Polygenic Architecture of Prostate Cancer Susceptibility (Genomics).

6. Conti, D.V., and et al. (2020). Multiethnic GWAS meta-analysis identifies novel variants and informs genetic risk prediction for prostate cancer across populations. Nature Genetics; to appear.
